# Updates to the Running Shoe and Injury Considerations

**DOI:** 10.7759/cureus.107402

**Published:** 2026-04-20

**Authors:** Thomas M Neubauer, Robert D Hill, Yao-Wen E Hu

**Affiliations:** 1 Family Medicine, Evans Army Community Hospital, Fort Carson, USA; 2 Family Medicine, Joint Base San Antonio, Lackland, USA; 3 Family Medicine, University of Massachusetts Chan Medical School, Worcester, USA

**Keywords:** clinical outcomes, footwear biomechanics, injury prevention, running injuries, running shoe

## Abstract

Elite athletes continue to break new records while running shoe design evolves, diverges, and incorporates new technologies. Shoe design is grossly split between minimalist, traditional, and maximalist shoes. Each shoe has a unique design with characteristics defined by the stack height, heel height, heel-to-toe drop, arch support, cushion, weight, and resulting biomechanics. Current epidemiology of running-related injuries correlates to experience level, weekly volume, and training progression, but the literature currently suggests there are limited effects from footwear. Although biomechanical research demonstrates the effects of footwear on the mechanical forces and stressors, clinically relevant outcomes are limited. Despite a lack of conclusive evidence, runners and clinicians often still perceive footwear as a key factor for injury prevention. This narrative review highlights the need for continued research into clinical outcomes and unbiased individualized footwear recommendations based on comfort, experience, and performance goals using current best-known practices.

## Introduction and background

Per World Athletics, as of 2025, the fastest marathon times for male and female competitors are two hours 35 seconds and two hours nine minutes 56 seconds, respectively [1,2]. These feats are made possible chiefly because of incredible individual physiology and years of unrelenting dedication to training. However, a portion of their success and performance seems to be tied to their running shoes [3]. Over the last 50 years, not only has running boomed, so has the variety of running shoes, making it challenging for runners and clinicians alike to stay updated on the different running footwear options. Often, patient education with regard to running shoes is not supported by scientific research [4]. Runners commonly assign more value to their internet searches and in-store analyses compared to advice and direction given by a healthcare professional [5]. This narrative review summarizes key elements of running shoe anatomy and evaluates recent evidence related to injury risk and footwear selection, with the goal of informing evidence-based clinical guidance for runners.

Review approach and sources

This manuscript provides a narrative overview of the existing literature concerning injuries and injury prevention related to running shoes. As a narrative review, the aim was not to be exhaustive in the manner of a systematic review, but to synthesize key themes and foundational concepts. Sources were identified through targeted searches in the PubMed databases, using search terms such as "running related injuries", "running shoes", "minimalist running shoes", and "maximalist running shoes". Additional articles were identified by reviewing the bibliographies of seminal papers in the field. The selection was guided by the author's professional judgment to include literature that shapes the understanding of the topic, with a focus on articles published in peer-reviewed journals within the last decade.

## Review

Shoe anatomy

The anatomy of the running shoe has undergone sweeping changes over the last 50 years. Early in the 1960s, companies made low-profile shoes with little to no cushioning involved. In contrast, modern running shoes have much more structure and design consideration. Each time a runner puts on a pair of shoes, the hope is that they will be able to run more efficiently in part because of the appropriate shoe. Understanding the components of a running shoe may assist clinicians in providing appropriate recommendations.

**Figure 1:**
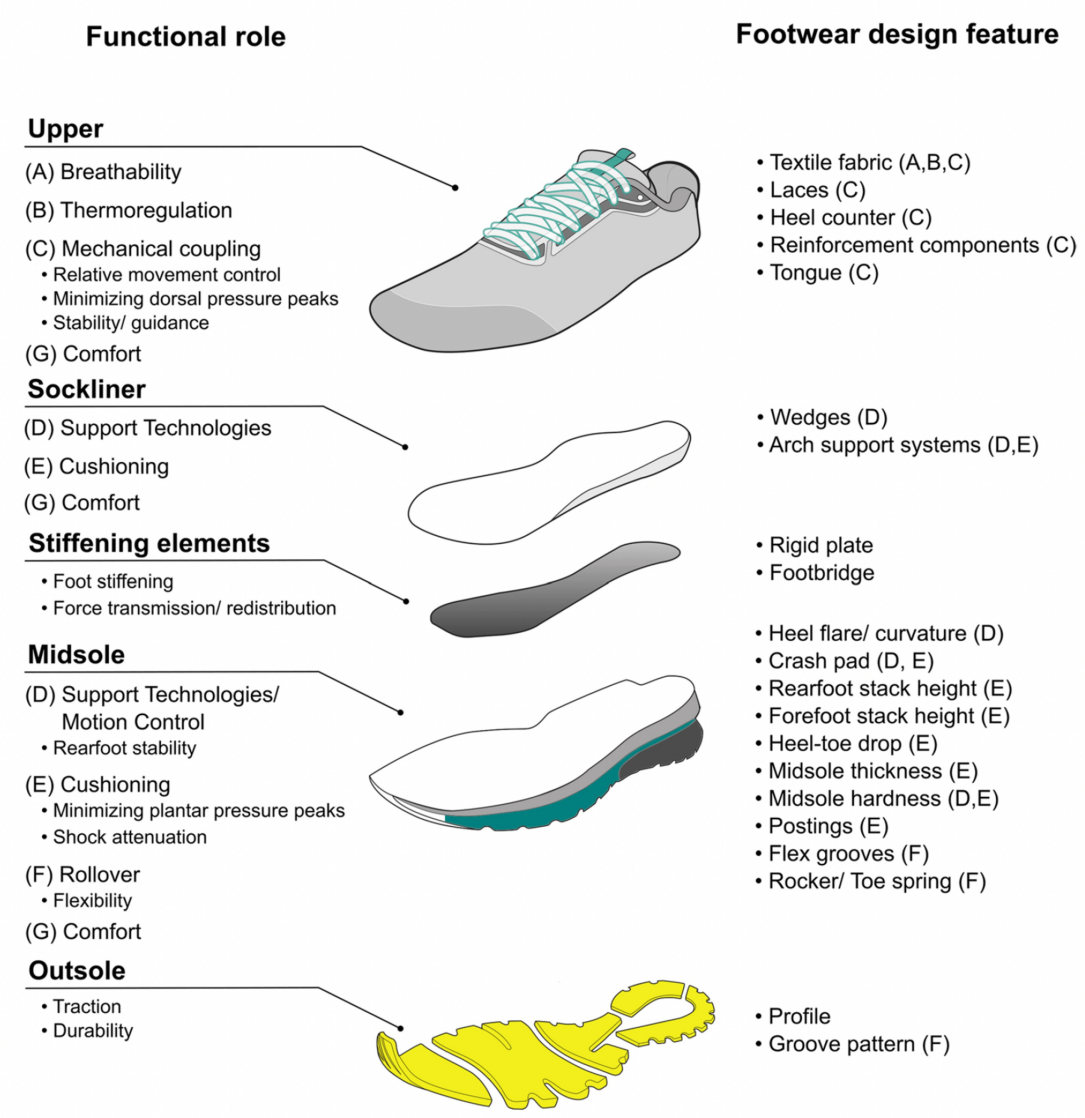
Running shoe anatomy. An assembly drawing of the various construction elements of common running footwear with their functional roles (left-hand side). Categorization of different footwear design features with the fulfilled function (right-hand side). Letters in parentheses link the functional sole of footwear to its modifiable footwear design feature(s). Figure Source: Mai et al., 2023 [7]; reproduced under the Creative Commons Attribution License (CC BY 4.0 Deed)

**Figure 2:**
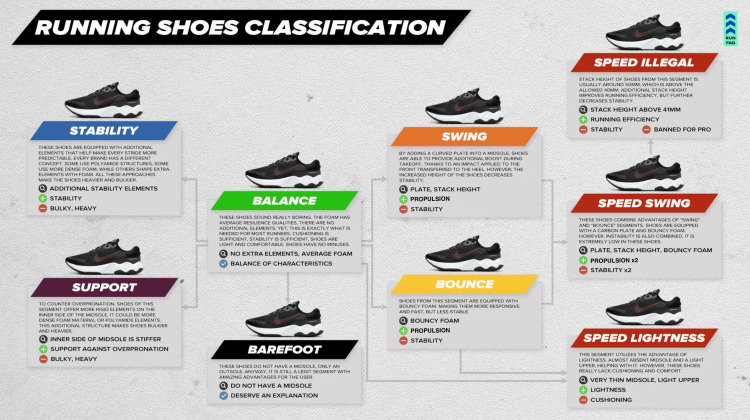
Running shoe classification. Running shoes can be organized into categories based on structural features (e.g., stability elements, midsole stiffness, stack height, foam composition) and functional outcomes (e.g., stability, propulsion, cushioning, lightness). Figure Source: RUN FAQ [8]; published with permission to use photo in a scientific medical journal publication

Running shoes, anatomy depicted in Figure 1 and classification displayed in Figure 2, are separated into two distinct parts: the upper and the sole. The upper is the portion of the shoe which includes the tongue, laces, sides, toe box, and heel counter. The sole, where the majority of research on running shoes have been focused, is the bottom part of the shoe that comes in contact with the plantar surface of the foot and the ground [5,6]. The sole is comprised of the insole, the midsole, and the outsole. The insole consists of the sock liner, which is often a thin pad resembling an orthotic, whereas the midsole is the portion of the shoe between the insole and the outsole. The thickness of the midsole, specifically in the heel, determines the stack height. Stack height differences, or lack thereof, produce minimalist, traditionalist, and maximalist shoes with different heel-to-toe drop parameters. Heel-to-toe drop, also referred to as "offset" or simply "drop", measures the difference in height between the heel and the forefoot of the shoe in millimeters [6]. For example, a 5-millimeter heel-to-toe drop means the heel midsole is 5-millimeters thicker than the forefoot midsole. 

Running shoe producers have generated individual innovations in shoe technology that have propelled modern running performance advancements known as advanced footwear technology (AFT) [9]. In general, AFT includes a midsole with lightweight foam that allows for energy return, a plate often made of carbon fiber embedded in the midsole which potentially decreases energy loss, and an outsole that curves upwards at the heel and at the toe potentially allowing for more spring [5].

Epidemiology of running injuries

It is estimated that between 19% and 79% of runners sustain a running-related injury (RRI) within a year, with an overall yearly incidence rate for distance running between 37% and 56% [10,11]. Although the skill level and time in training vary widely among runners, both professional and recreational runners alike share similar RRIs, most commonly injuring the knee, lower leg, foot, and upper leg [11,12]. Toresdahl et al. found that among 675 first-time runners of the New York City marathon, 59% experienced at least one RRI during training or during the race, ranging from bone stress injuries to Achilles tendinosis and multiple other lower extremity injuries [10]. Furthermore, as running mileage increases per week, the incidence of RRI increases proportionally, while previous injuries increase the risk of recurrence [10]. Despite the risk of RRI, running is generally safe with important cardiovascular benefits; further research is needed for those with chronic diseases [13].

Biomechanical shoe design and injuries

When a runner plants the heel into the ground after the double-float phase, the body is exposed to vertical forces of 2-3 times the body weight in the ipsilateral lower extremity [14]. If inappropriately managed, resultant repetitive loading is thought to be the impetus to common RRIs including bone stress injury, plantar fasciopathy, and Achilles tendinopathy, among others [14,15]. However, attempts to fit running shoes to plantar foot shape in military basic training in hopes of reducing RRI have not been proven to reduce injury risk [16,17]. Interestingly, Sun et al. found that a stiffer running shoe may positively affect athletic performance, while a softer, thicker midsole may decrease RRI [5], but more research is needed regarding the biomechanical effects of heel flare, shoelace, and heel cups on running [6]. High-quality evidence linking individual biomechanics to RRI is unfortunately lacking [17], although Jiang et al. [18] noted that novice runners display greater changes in joint angles, joint moments, and ground reaction forces compared to experienced runners [6]. Despite AFT encouraging runners into a forefoot or midfoot strike pattern, causing less subtalar eversion, and resulting in different biomechanical forces experienced by the foot, further research is still needed to understand AFT's benefits for runners [18-20].

Cushioning

Cushioning is a core feature of running shoe design that affects impact forces, running mechanics, and comfort, with potential implications for running injuries. The running shoe industry markets both high and minimal cushioning options, each having differing effects on running biomechanics. Malisoux et al. evaluated the impact of running shoe cushioning on injury rates, finding higher injury risk among light runners using hard shoes [21]. Two other secondary analyses examining possible biomechanical causes for injuries showed a decrease in the magnitude and frequency of impact forces in the soft shoe [22] and that shoe stiffness is related to the increased mechanical burden on the ankle and hip [23]. However, vertical impact peak forces, vertical instantaneous loading rate, and ankle eversion velocity were not associated with increased injury risk [23]. These biomechanical studies suggest that the protective effects of shoe cushioning were not uniform, with lighter runners experiencing more injuries in harder shoes [21]. Despite lower impact forces, a stiff shoe may increase the risk of injuries due to the frequency of impact forces and altered joint loading patterns [22,23].

Heel

Heel design in footwear has two primary characteristics (heel height and heel-to-toe drop) that are commonly found in many running shoes and affect running mechanics. Biomechanical analysis of heel height revealed that minimalist shoes without heel elevation increased frontal plane ankle torque compared to barefoot running, whereas higher heels reduced torque due to postural changes [24]. Heel height also increased ankle plantar flexion velocity at heel strike [24]. Yawar and Lieberman found an unexpected, complex, and nonlinear relationship between heel height and ankle mechanics, highlighting the complexity of heel height in running and the need for adaptation [24]. However, Malisoux et al. found no significant difference in injury risk for standard cushioned running shoes with 10-millimeter, 6-millimeter, and 0-millimeter heel-to-toe drops yet regular runners had a higher risk for injury in 6-millimeter and 0-millimeter heel-to-toe drop shoes compared to occasional runners [25]. Malisoux et al. also found that, in occasional runners, there was no increased risk of RRI associated with using either 6-millimeter or 0-millimeter low-drop shoes [25]. Furthermore, Gu et al. showed that although there was no significant difference in perceived pain, negative heel shoes decreased peak patellofemoral joint stress among other factors [26]. Further strides in research are needed to evaluate the long-term adaptation and performance of heel height in running. 

The minimalist versus maximalist debate

As the minimalist shoe movement gathered mainstream attention in 2009, footwear companies began developing minimalist shoes that focused on a natural running pattern, characterized by lower mass, reduced cushioning, low heel-to-toe drop, and increased ground feel. Rixe et al. reviewed the history of running since humans began walking 4.4 million years ago, noting the end of the barefoot era 30,000 years ago and that no clinical studies have demonstrated superiority in reducing injuries despite known biomechanical differences [27-29].

Alterations in running mechanics are associated with the transition to forefoot striking while using minimalist running shoes. Yang et al. reported that gait retraining resulted in a 78% conversion rate, with participants transitioning from rearfoot to forefoot striking, reducing loading rates and peak impact forces compared to those who received minimalist shoes without gait retraining [28]. Davis et al. further detailed the biomechanical effects of minimalist shoes and a forefoot strike pattern, leading to plantar fascia and Achilles tendon loading with a spring-like function and increasing intrinsic and extrinsic foot muscular strength to promote soft landings [29].

Comparing minimalist and conventional running shoes, Fuller et al. showed that minimalist shoes were associated with an increased risk of injury in runners weighing more than 71.4 kilograms [30]. Maximalist running shoes are characterized by a high stack height, thick midsole cushioning, and soft rockered soles with a neutral or low heel-to-toe drop of 4-8 millimeters. Pollard et al. demonstrated biomechanical changes leading to increased impact forces and loading rates with maximalist running shoes [31]. Furthermore, Hannigan and Pollard compared maximalist, traditional, and minimalist shoes, showing increased loading rate when comparing minimal to maximal shoes, while peak eversion was greater in both the maximal and minimal shoes compared to the traditional shoe [32]. The maximalist shoe also had greater eversion duration and eversion at toe-off [32].

Running shoes, injury risk, and injury prevention

Recent literature suggests prior injury, over-training, and abnormal biomechanics and running alignment along with high load or impact as contributors to RRIs such as stress fractures, plantar fasciitis, patellofemoral syndrome, and chronic exertional compartment syndrome [33,34]. Interestingly, runners perceive footwear to be important for reducing the rate of RRIs, despite most data showing that footwear is unrelated to the risk of injury [4,35-37]. Furthermore, although runners may perceive increased cushioning in footwear to be preventative and footwear breakdown to be deleterious, these perceptions do not appear to be substantiated by recent evidence [36]. The need for further research with regard to running shoes and their relations to injury risk and prevention is further corroborated in military studies showing mixed evidence regarding running shoes and lower extremity injuries [38,39].

Overuse injuries

While the evidence does not correlate the risk of specific overuse running injuries to running footwear, recent studies have shown that specific footwear influences biomechanical factors and alignment. Additionally, Mai et al. showed that specific alterations to running shoes may help decrease individual biomechanical risk factors for injury [7]. Malisoux et al. and Willems et al. also showed that motion-controlled running shoes led to fewer pronation-related injuries in runners with foot pronation [40,41], but the understanding of the true pathophysiology is still limited. Although potentially beneficial modifications in biomechanics and alignment are linked to running shoe alterations, further research is needed to evaluate specific injury risk for overuse injuries with respect to running shoes instead of relying on extrapolation and inference.

Bone stress injuries

Specific footwear has not been shown to prevent bone stress injuries in runners either. Lavigne et al. found that while foot orthoses may potentially prevent bone stress injuries in the military, it is still unknown whether footwear could have any benefits [42]. While increased cushioning may intuitively postulate decreased impact and vertical forces sustained in the lower extremity, there is no evidence that substantiates this theory. With changes in footwear, it is also important to recognize that drastic and rapid changes in running shoes may increase the risk of injury [43]. Tenforde et al. reported a series of bone stress injuries in runners using carbon fiber plating running shoes [44], suggesting extra caution be displayed when recommending running footwear and highlighting the need for further research.

Correlations to clinical practice

While literature does not support specific injury prevention with respect to running footwear, clinical providers and runners themselves still believe running shoes reduce RRIs. But, Ayisi et al. reported that although 89% of clinical providers feel footwear prescriptions reduce the risk of running injuries, most clinicians do not discuss running footwear with patients due to self-reported lack of knowledge and allotted clinical time [45]. It is unclear why clinicians perceive preventive benefits of running footwear despite minimal evidence, but the admitted lack of knowledge is problematic. 

Running shoe selection

Despite both runners and clinicians believing a running shoe prescription reduces injuries, currently, there is no best shoe to prevent injury [4,34-37,45]. Relph et al. concluded that current evidence does not support the idea that specific shoe types reduce running injury risk, emphasizing the need for better study designs, standardization of shoe features, and uniform definitions of injuries [37]. Furthermore, Mai et al. recommended against tailoring footwear design features to individual runners [7]. However, avoiding harder and stiffer shoes in lightweight runners or minimalist shoes in runners weighing over 71 kilograms may be beneficial for comfort and the possible reduction of injury [21,30]. Nonetheless, the history of previous injury, transition between shoes, weekly mileage, training program progression, running experience, and nutrition should still remain as pillars for running injury prevention [11,44,46]. Running shoe selection requires several considerations including comfort, runner experience, training goals, injury history, body mass, cost, and availability. Improving training conditions with adequate nutrition, recovery, and manageable stress also allows for adaptations to various environments [47,48].

Interestingly, Honert et al. created classifications and shoe recommendations for runners along with five footwear properties important for all running levels, included in Table 1 [49]. The classifications, recommendations, and specific shoe properties including upper breathability, forefoot bending stiffness, heel-to-toe drop, torsional bending stiffness, and crash pad [49] may offer a framework for future research.

**Table 1:** Running level definitions and corresponding priorities for footwear design. These definitions were refined by the Delphi study participants through the three rounds of feedback. The * indicates regular running experience defined as running at least once per week. The $ indicates that elite runners with faster race times than high-caliber runners were not considered since they represent a small percentage of the population and may require individual running footwear recommendations. Table Source: Honert et al., 2020 [49]

	Level 1: novice	Level 2: recreational	Level 3: high caliber
Running experience	Less than one year of regular* running experience	More than one year of regular* running experience	More than three years of regular* running experience
Running habits	0-3 sessions	1-5 sessions/week	>4 sessions/week
	5-20 km/week	15-50 km/week	> 50 km/week
Running performance (example times are for male runners age 18-34)	5 km time >30 min OR	5 km time >21 min OR	5 km time 15-20 min^$ ^OR
	10 km time >60 min	10 km time >42 min OR	10 km time 30-42 min^$^ OR
	No marathon racing	Marathon time 3-4.5 hours	Marathon time <3 hours
Running motivation (ordered according to importance)	Improve general health	Improve general health	Competition
	Enjoyment	Enjoyment	Improve general health
	Weight management	Team affiliation	Enjoyment
Priorities for footwear design (from high to low)	(1) Improve comfort	(1) Improve comfort	(1) Improve performance
	(2) Reduce injury risk	(2) Reduce injury risk	(2) Reduce injury risk
	(3) Improve performance	(3) Improve performance	(3) Improve comfort

## Conclusions

Professional and recreational runners continue to rely on the technology of the running shoe to pursue improved athletic performance. Recent experimentation with the complex interaction of shoe materials and shape has gained traction, while barefoot or minimalist shoes have re-emerged. The basic anatomy of the running shoe may seem simple, but the details regarding the materials and shape create many individualized and specialized facets of the shoe that are difficult to compare and study. RRIs continue to occur in over 50% of runners and are linked to a history of previous injury, running experience, weekly mileage, and training progression. Despite current literature demonstrating various running footwear's differing biomechanical effects, there is no correlation between running shoes and injury prevention, and further research is needed. Future research is needed to clarify the relationship between footwear characteristics and injury risk, but current evidence supports an individualized, comfort-guided approach to footwear selection.

## References

[1] Kiptum K (2024). Ratified: Kiptum's world marathon record. http://worldathletics.org/news/press-releases/ratified-world-marathon-record-kelvin-kiptum.

[2] Chepng’etich Chepng’etich, R R (2025). Ratified: world records for Chebet, Duplantis, McLaughlin-Levrone, Chepngetich and Kawano. http://worldathletics.org/news/press-releases/ratified-world-records-chebet-duplantis-mclaughlin-levrone-chepngetich-kawano.

[3] Muniz-Pardos B, Sutehall S, Angeloudis K, Guppy FM, Bosch A, Pitsiladis Y (2021). Recent improvements in marathon run times are likely technological, not physiological. Sports Med.

[4] Bermon S (2021). Evolution of distance running shoes: performance, injuries, and rules. J Sports Med Phys Fitness.

[5] Agresta C, Giacomazzi C, Harrast M, Zendler J (2022). Running injury paradigms and their influence on footwear design features and runner assessment methods: a focused review to advance evidence-based practice for running medicine clinicians. Front Sports Act Living.

[6] Sun X, Lam WK, Zhang X, Wang J, Fu W (2020). Systematic review of the role of footwear constructions in running biomechanics: implications for running-related injury and performance. J Sports Sci Med.

[7] Mai P, Robertz L, Robbin J (2023). Towards functionally individualised designed footwear recommendation for overuse injury prevention: a scoping review. BMC Sports Sci Med Rehabil.

[8] (2025). Running shoes classification. http://www.runfaq.com/shoes_classification.

[9] Knopp M, Muñiz-Pardos B, Wackerhage H, Schönfelder M, Guppy F, Pitsiladis Y, Ruiz D (2023). Variability in running economy of Kenyan world-class and European amateur male runners with advanced footwear running technology: experimental and meta-analysis results. Sports Med.

[10] Toresdahl B, McElheny K, Metzl J, Kinderknecht J, Quijano B, Ammerman B, Fontana MA (2022). Factors associated with injuries in first-time marathon runners from the New York City marathon. Phys Sportsmed.

[11] van Gent RN, Siem D, van Middelkoop M, van Os AG, Bierma-Zeinstra SM, Koes BW (2007). Incidence and determinants of lower extremity running injuries in long distance runners: a systematic review. Br J Sports Med.

[12] Taunton JE, Ryan MB, Clement DB, McKenzie DC, Lloyd-Smith DR, Zumbo BD (2002). A retrospective case-control analysis of 2002 running injuries. Br J Sports Med.

[13] Braschler L, Nikolaidis PT, Thuany M (2025). Physiology and pathophysiology of marathon running: a narrative review. Sports Med Open.

[14] Wang IL, Graham RB, Bourdon EJ, Chen YM, Gu CY, Wang LI (2020). Biomechanical analysis of running foot strike in shoes of different mass. J Sports Sci Med.

[15] Milner CE, Ferber R, Pollard CD, Hamill J, Davis IS (2006). Biomechanical factors associated with tibial stress fracture in female runners. Med Sci Sports Exerc.

[16] Knapik JJ, Brosch LC, Venuto M (2010). Effect on injuries of assigning shoes based on foot shape in air force basic training. Am J Prev Med.

[17] Knapik JJ, Trone DW, Swedler DI (2010). Injury reduction effectiveness of assigning running shoes based on plantar shape in Marine Corps basic training. Am J Sports Med.

[18] Jiang X, Xu D, Fang Y, Bíró I, Baker JS, Gu Y (2023). PCA of running biomechanics after 5 km between novice and experienced runners. Bioengineering (Basel).

[19] Kim H, Ahn J (2025). Technologically advanced running shoes reduce biomechanical factors of running related injury risk. Sci Rep.

[20] Leporace G, Guadagnin EC, Carpes FP, Gustafson J, Gonzalez FF, Chahla J, Metsavaht L (2026). The search for the holy grail in running biomechanics: is there an ideal movement profile for minimizing mechanical overload?. Sports Health.

[21] Malisoux L, Delattre N, Urhausen A, Theisen D (2020). Shoe cushioning influences the running injury risk according to body mass: a randomized controlled trial involving 848 recreational runners. Am J Sports Med.

[22] Malisoux L, Gette P, Backes A, Delattre N, Cabri J, Theisen D (2021). Relevance of frequency-domain analyses to relate shoe cushioning, ground impact forces and running injury risk: a secondary analysis of a randomized trial with 800+ recreational runners. Front Sports Act Living.

[23] Malisoux L, Gette P, Backes A, Delattre N, Theisen D (2023). Lower impact forces but greater burden for the musculoskeletal system in running shoes with greater cushioning stiffness. Eur J Sport Sci.

[24] Yawar A, Lieberman DE (2024). Effects of shoe heel height on ankle dynamics in running. Sci Rep.

[25] Malisoux L, Chambon N, Urhausen A, Theisen D (2016). Influence of the heel-to-toe drop of standard cushioned running shoes on injury risk in leisure-time runners: a randomized controlled trial with 6-month follow-up. Am J Sports Med.

[26] Gu Y, Zheng Z, Zeng Q, Yang C, Song Y, Wan X (2024). Acute effects of negative heel shoes on perceived pain and knee biomechanical characteristics of runners with patellofemoral pain. J Foot Ankle Res.

[27] Rixe JA, Gallo RA, Silvis ML (2012). The barefoot debate: can minimalist shoes reduce running-related injuries?. Curr Sports Med Rep.

[28] Yang Y, Zhang X, Luo Z, Wang X, Ye D, Fu W (2020). Alterations in running biomechanics after 12 week gait retraining with minimalist shoes. Int J Environ Res Public Health.

[29] Davis IS, Rice HM, Wearing SC (2017). Why forefoot striking in minimal shoes might positively change the course of running injuries. J Sport Health Sci.

[30] Fuller JT, Thewlis D, Buckley JD, Brown NA, Hamill J, Tsiros MD (2017). Body mass and weekly training distance influence the pain and injuries experienced by runners using minimalist shoes: a randomized controlled trial. Am J Sports Med.

[31] Pollard CD, Ter Har JA, Hannigan JJ, Norcross MF (2018). Influence of maximal running shoes on biomechanics before and after a 5k run. Orthop J Sports Med.

[32] Hannigan JJ, Pollard CD (2020). Differences in running biomechanics between a maximal, traditional, and minimal running shoe. J Sci Med Sport.

[33] Altman AR, Davis IS (2012). Barefoot running: biomechanics and implications for running injuries. Curr Sports Med Rep.

[34] Burke A, Dillon S, O'Connor S, Whyte EF, Gore S, Moran KA (2023). Aetiological factors of running-related injuries: a 12 month prospective "Running Injury Surveillance Centre" (RISC) study. Sports Med Open.

[35] Dhillon GK, Hunt MA, Reid AL, Esculier JF (2020). What are the perceptions of runners and healthcare professionals on footwear and running injury risk?. BMJ Open Sport Exerc Med.

[36] Bachand R, Bazett-Jones DM, Esculier JF, Fox C, Norte GE, Garcia MC (2024). The dogma of running injuries: perceptions of adolescent and adult runners. J Athl Train.

[37] Relph N, Greaves H, Armstrong R (2022). Running shoes for preventing lower limb running injuries in adults. Cochrane Database Syst Rev.

[38] Knapik JJ, Pope R, Orr R, Grier T (2015). Injuries and footwear (part 1): athletic shoe history and injuries in relation to foot arch height and training in boots. J Spec Oper Med.

[39] Helton GL, Cameron KL, Zifchock RA, Miller E, Goss DL, Song J, Neary MT (2019). Association between running shoe characteristics and lower extremity injuries in United States military academy cadets. Am J Sports Med.

[40] Malisoux L, Chambon N, Delattre N, Gueguen N, Urhausen A, Theisen D (2016). Injury risk in runners using standard or motion control shoes: a randomised controlled trial with participant and assessor blinding. Br J Sports Med.

[41] Willems TM, Ley C, Goetghebeur E, Theisen D, Malisoux L (2021). Motion-control shoes reduce the risk of pronation-related pathologies in recreational runners: a secondary analysis of a randomized controlled trial. J Orthop Sports Phys Ther.

[42] Lavigne A, Chicoine D, Esculier JF, Desmeules F, Frémont P, Dubois B (2023). The role of footwear, foot orthosis, and training-related strategies in the prevention of bone stress injuries: a systematic review and meta-analysis. Int J Exerc Sci.

[43] Malisoux L, Theisen D (2020). Can the "appropriate" footwear prevent injury in leisure-time running? Evidence versus beliefs. J Athl Train.

[44] Tenforde A, Hoenig T, Saxena A, Hollander K (2023). Bone stress injuries in runners using carbon fiber plate footwear. Sports Med.

[45] Ayisi J, Gregorek S, Lu E, Walrod B (2025). 2025 AMSSM research oral poster presentations. Clin J Sport Med.

[46] van der Worp MP, ten Haaf DS, van Cingel R, de Wijer A, Nijhuis-van der Sanden MW, Staal JB (2015). Injuries in runners; a systematic review on risk factors and sex differences. PLoS One.

[47] Mountjoy M, Sundgot-Borgen JK, Burke LM (2018). IOC consensus statement on relative energy deficiency in sport (RED-S): 2018 update. Br J Sports Med.

[48] Kellmann M, Bertollo M, Bosquet L (2018). Recovery and performance in sport: consensus statement. Int J Sports Physiol Perform.

[49] Honert EC, Mohr M, Lam WK, Nigg S (2020). Shoe feature recommendations for different running levels: a Delphi study. PLoS One.

